# Analysis of 126 hospitalized elder maxillofacial trauma victims in central China

**DOI:** 10.4317/medoral.20551

**Published:** 2015-04-10

**Authors:** Rui Li, Rui Zhang, Wenlu Li, Fei Pei, Wei He

**Affiliations:** 1 Department of Stomatology, the First Affiliated Hospital of Zhengzhou University, Zhengzhou University, China; 2 Oral and Maxillofacial Surgery, Zheng Zhou Stomatologic Hospital, China

## Abstract

**Background:**

The aim of this study was to analyzed the characteristics and treatment of maxillofacial injuries in the elder patients with maxillofacial injuries in central China.

**Material and Methods:**

We retrospectively analyzed the characteristics and treatment of maxillofacial injuries in the patients over the age of 60 to analyze the trends and clinical characteristics of maxillofacial trauma in elder patients from the First Affiliated Hospital of Zhengzhou University (from 2010 to 2013) in central China and to present recommendations on prevention and management.

**Results:**

Of the 932 patients with maxillofacial injuries, 126 aged over 60 years old accounting for 13.52% of all the patients (male:female, 1.74:1; mean age, 67.08 years old). Approximately 52% of the patients were injured by falls. The most frequently observed type of injuries was soft tissue injuries (100%), followed by facial fractures (83.05%). Of the patients with soft tissue injuries, the abrasions accounted the most, followed by lacerations. The numbers of patients of midface fracture (60 patients) were almost similar to the number of lower face fractures (66 patients). Eighty two patients (65.08%%) demonstrated associated injuries, of which craniocerebral injuries were the most prevalent. One hundred and four patients (82.54%) had other systemic medical conditions, with cardiovascular diseases the most and followed by metabolic diseases and musculoskeletal conditions. Furthermore, the study indicated a relationship between maxillofacial fractures and musculoskeletal conditions. Only 13 patients (10.32%) sustained local infections, of whom had other medical conditions. Most of the facial injuries (85.71%) in older people were operated including debridement, fixing loose teeth, reduction, intermaxillary fixation and open reduction and internal fixation (ORIF).

**Conclusions:**

Our analysis of the characteristics of maxillofacial injuries in the elder patents may help to promote clinical research to develop more effective treatment and possibly prevent such injuries.

**Key words:** Maxillofacial, trauma, elderly, characteristics, treatment.

## Introduction

Maxillofacial injuries are a serious health problem worldwide (1). As a constituent part of general trauma, oral and maxillofacial injuries do not typically constitute a direct threat to life, but these injuries may be considered more serious than injuries to other body parts due to the importance of the appearance of the face and emotional distress that accompanies these injuries. Maxillofacial trauma in the general population has been well studied (2-4). Reports also exist concerning maxillofacial injuries in children (5,6). However, very few studies have investigated maxillofacial fractures specifically in the elderly sub population. In recent years, trauma in the elderly has been increasing due to the increased life span with advances in medicine, resulting in a greater percentage of elder people in the population. Census data from the Chinese Bureau of Statistics including the population pyramid for China show that for the past decade the percentage of people aged over 60 has risen (10.33%-13.26%), which indicates China has come into an aging society (7). Trauma has a greater physical impact on the elder age people because of their decreased physical reserves and age-related coexisting conditions, such as cardiovascular disease, poor eyesight, osteoporosis, reduced muscle mass, arthritis, and so on (8). Besides, many elder patients are associated with organ system dysfunctions, such as ischaemic heart disease and dysrhythmias (9). Despite the morbidity associated with maxillofacial trauma among elder people, little research regarding it exists, especially systemic research. Therefore, an understanding of the etiology, pattern, and consequences of maxillofacial injuries in the elderly is essential to successfully prevent maxillofacial injuries of these patients and also to develop more effective treatment.

He’nan province locates in the middle of China with a population of more than 100 million inhabitants. It is the traffic center of China and has a large transient population. Because of the geographic and demographic features, He’nan province is an area prone to injuries, especially by traffic accident and assault. So far, we have not found any reports about the status of maxillofacial injuries in this region.

The purpose of the present study was to analyze the trends and clinical characteristics of maxillofacial trauma in elder patients in He’nan province and to present recommendations on prevention and management.

## Patient and Methods

We made a 3-year prospective observational study (2010–2013) of 932 patients with maxillofacial trauma who were presented to the First Affiliated Hospital of Zhengzhou University (the largest medical center of He’nan province). Patients’ information was collected based on the Hospital Information System (HIS), including age, gender, mechanism of injury, frequency and type of injury (dentoalveolar injuries, soft tissue injuries, and facial fractures), associated injuries (craniocerebral injury, important organ injury, extremity injury and others), infections, and treatment modality. In case of that, the same patient was injured more than once during the study period, all injuries were registered, except for repeated visits or admissions for the same injury.

Distribution of soft tissue injuries was recorded using the MCFONTZL system developed by Lee *et al*. (10) with modifications; the authors added scalp as a new site of injury according to our previous study (11). Every patient with maxillofacial injuries had been given proper examinations such as X-rays or computed tomography to make clinical diagnosis clear and exact. Data were analyzed by the maxillofacial trauma database and Microsoft Access 2000 software.

The protocol of the study was approved by the Ethics Committee and the Institutional Review Board (IRB) of the First Affiliated Hospital of Zhengzhou University, Zhengzhou University, China. Each subject in the project signed a detailed informed consent form.

The chi-square test was used to test the association between two categorical variables or factors (such as gender, cause). *P* value <0.05 was considered to be statistically significant. All analyzes were performed with SPSS 16.0 software (Inc., Chicago, IL, USA).

## Results

- General characteristics

Of the 932 patients, 126 aged over 60 years old, accounting for about 13.52% of all the maxillofacial injuries. The elderly maxillofacial injury patients consisted of 80 men (63.49%) and 46 women (36.51%). To further investigate the association between age and injuries characteristics, we divide these patients into 5 subgroups: 60s-69s, 70s-79s, 80s-89s, 90s-99s and over 100s. The age distribution of male and female patients is listed in  Table 1. The male to female ratio was 1.74:1 and the mean age of these patients was 67.08 years old (range, 60–86 years). The majority of male patients were between 60 and 69 years old, while female patients exhibited a similar distribution.

**Table 1:**
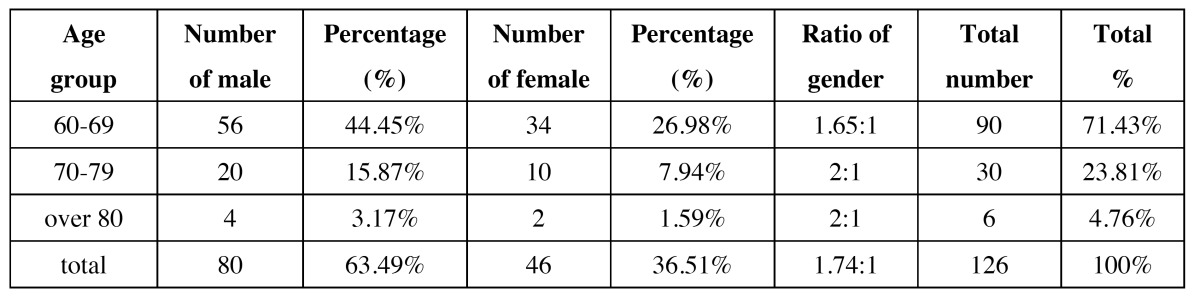
The age and gender distribution of the elder patients.

The monthly distribution peaked in the summer (July, 9.5%), but revealed further peak incidences during December (8.8%), January (9%), and February (8.9%). November figures were lowest.

- Injury Mechanism

Our study showed that the most common cause of facial injuries among older people was falls (52.38%), followed by motor vehicle accidents (33.33%). Other types of injury mechanism accounted for a less portion. There is no relationship between age and injury mechanism (data not shown).

- Types of Maxillofacial Injuries

The most frequently observed type of injuries was soft tissue injuries (100%), followed by facial fractures (83.05%). One hundred patients suffered combined injuries, in which 98 patients suffered soft tissue injuries and facial fractures; 12 patients suffered soft tissue and dentoalveolar injuries; and 10 patients suffered soft tissue, dentoalveolar, and facial fractures. Twenty-six patients sustained simple soft tissue injuries.

**Figure 1:**
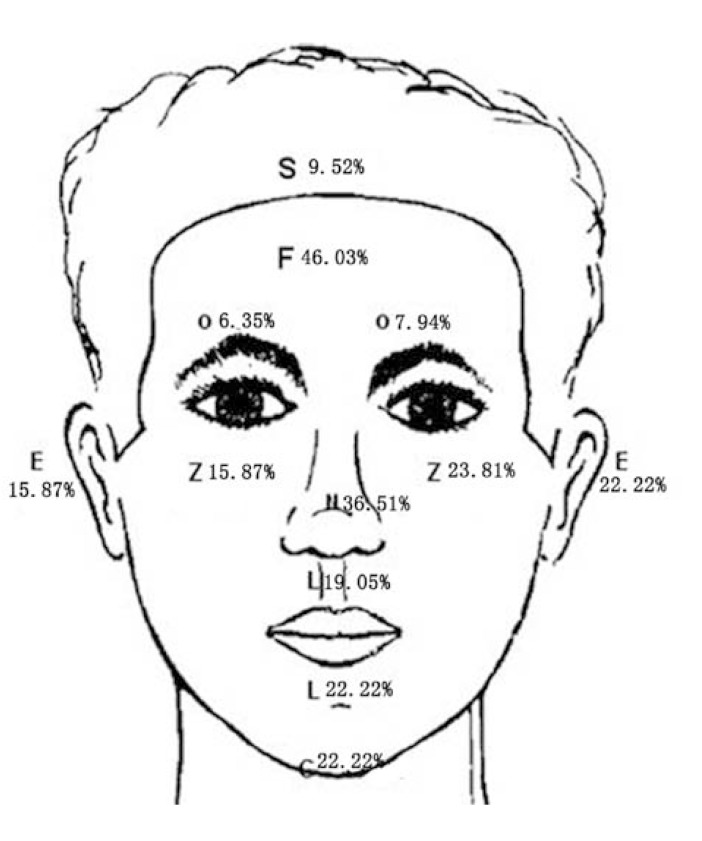
Overall distribution of soft-tissue injuries.

The soft tissue injuries were divided into laceration, abrasion, avulsion, contusion, and penetrating wounds. Of the 126 maxillofacial injury patients, 104 (82.54%) were abrasions, followed by lacerations (57.15%). Patients with avulsion or contusion or penetrating accounted for a lesser proportion (data not shown). According to figure 1, the location of this type of trauma was more frequent in the forehead (F;46.03%) and lip (L;41.27%). This was followed by the injuries at the zygomatic region (Z; 39.68%), buccal region (B;38.12%), nose (N;36.51%), and chin (C;22.22%). The number of injuries of periorbital area (O; 14.29%) and ear (E) were almost the same (15.87%). The injuries of scalp accounted for the least (9.52%).

**Figure 2:**
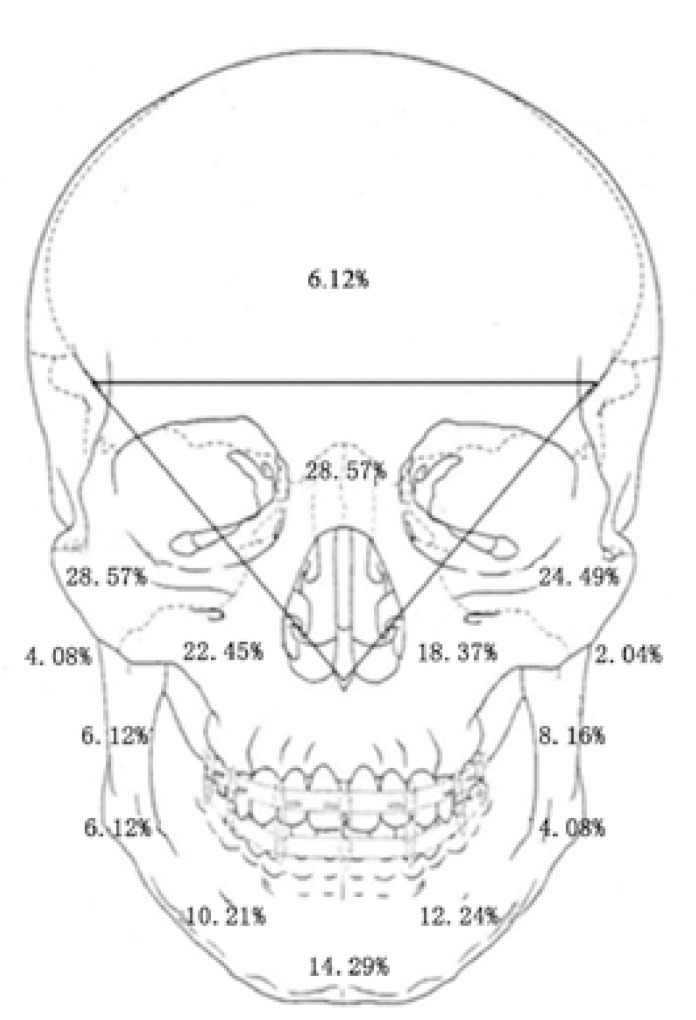
Distribution of facial fractures.

The facial fractures were classified according to anatomical site and to simplify the analysis, we further divided the locations into three parts: frontal bone, mid face, and mandible. The mid face consisted of maxilla, the nasal-orbital-ethmoidal region, and zygomatic bone. In the present study, 98 patients [66 males (67.35%), 32 females (32.65%)] with 192 maxillofacial fractures were analyzed. Of the 98 patients, 6 patients were with fontal bone fracture, 60 with mid face fracture and 66 with mandibular fracture. Figure 2 displays the distribution of facial fractures (the patients accounting for the whole patients sustaining facial fractures). Among the mid face fractures, zygomatic bone was significantly the most common (52 of 98 patients with zygomatic bone fractures, 53.06%). That was closely followed by maxillary bone (40.82%) and NOE (28.57%). The mandible accounted for 67.35% of injuries (body 22.45%, angle 10.20%, symphysis 14.29%, ramus 14.29%, condylar fracture 6.12%). Frontal bone (6.12%) had a less proportion with a percentage of 6.12%. The fractures often occurred in at least two sites.

- Associated Injuries and Systemic Diseases

**Figure 3:**
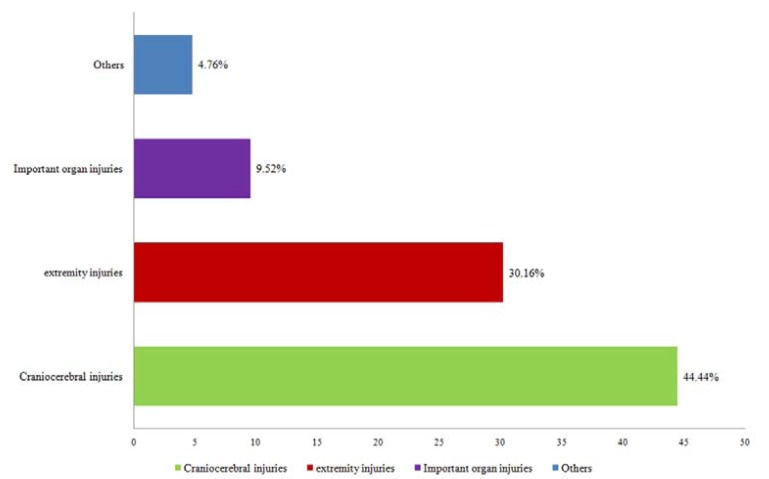
Distribution of associated injuries.

Injury to other sites of the body occurred in 82 patients (65.08%). Craniocerebral injuries (44.44%) were the most, followed by extremity injuries (30.16%) and important organ injuries, such as lung and abdominal (19.05%). Other parts, such as cervical spine injuries and eye injuries accounted for a less percentage, with a proportion of 14.29% (Fig. 3). There is no relationship between associated injuries and maxillofacial injuries types.

Of these elder patients, 104 patients had other medical conditions (82.54%). The most common condition was cardiovascular disease. It was followed by metabolic diseases and musculoskeletal conditions including osteoporosis, which were thought to an independent risk factor for maxillofacial fractures (12). Our study showed there was a relationship between maxillofacial fractures and musculoskeletal conditions ( Table 2).

**Table 2:**
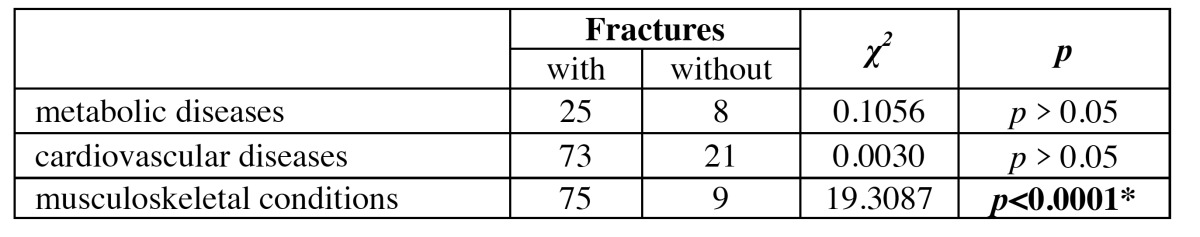
The relationship between maxillofacial fractures and systemic diseases. Musculoskeletal conditions could be a risk factor for maxillofacial fractures.

- Infections

None of the patients suffered general infections, but 13 patients (10.32%) sustained local infections. During these patients with local infections, 10 had other medical conditions. There was no relationship between infections and systemic diseases (data not shown).

- Treatments

Overall, 85.71% of the facial injuries in older people were operated including debridement, fixing loose teeth, reduction, inter maxillary fixation and open reduction and internal fixation (ORIF).

(1) to the soft tissue injuries: debridement was given and only 13 patients lead to a later local infection; (2) to the facial fractures: the majority of the patients (77.55%) were treated surgically, such as ORIF. The mandibular fractures had the greatest proportion treated surgically. Most orbital wall fractures were managed conservatively. With age increasing, the rate of surgical treatment decreased (*p*<0.05) ( Table 3).

**Table 3:**
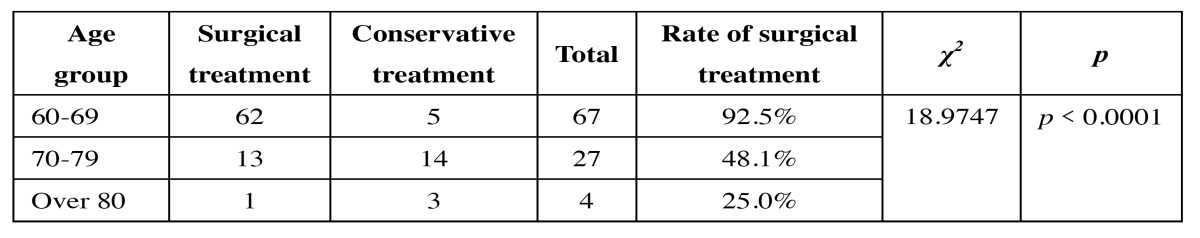
The treatment in different age group with facial fractures. As age increased, the rate of surgical treatment decreased.

## Discussion

The present study retrospectively analyzed the data of maxillofacial injuries in elderly patients and showed the trends and characteristic features of maxillofacial injuries in these patients. Maxillofacial injuries in elder patients have been increasing in recent years and reached nearly 15% of all maxillofacial injuries. These results are considered to reflect the increased life span, increased elderly population, and more active lifestyle of the older population (13,14).

Regarding gender, studies in the world literature demonstrate that men are more affected than women, and this predominance keeps constant throughout time (15). However, the data in the present study demonstrated that prevalence of injury did not differ by gender, with the overall male to female ratio 1:1.07. This figure was accordant with reports about maxillofacial injuries in the elder people by Yamamoto in Japan (16) and Jamieson in Australia (13).

The etiology of maxillofacial injuries varies from one country to another and even from one region to another within the same country, and it was influenced by the prevailing socioeconomic, cultural and environmental factors (17). Investigators in countries such as Finland, the United States, and Sweden have found that assault represent the most common cause of maxillofacial injuries (18-20). More reports suggested that traffic accident was the most common cause of maxillofacial injuries in China (17,21). In the present study, we found that falls and motor vehicle accidents were the leading causes of facial trauma among older people, which was in accordance with other studies (9,22). This finding indicates that patients younger than 60 years old are still socially active and might be involved in traffic accidents. The reason may be that most of studies investigated the patients of all age groups, and few focused on the elder people in China. Furthermore, as many Chinese over the age of 60 are still working and drive long distances to work, especially in the rural areas. Injuries from assaults and sports activities rarely occurred.

In the terms of injury type, all the patients suffered soft tissue injuries. Furthermore, the percentage of maxillofacial fractures was relatively high. The percentages added up to greater than 100%. That was because most patients suffered more than one type of injury. Our data was similar to the findings of several studies (23,24), but different from another one (25) that reported dental injuries as the most common type of trauma. In our study, dentoalveolar injuries were very rare owing to the absence of teeth in the area of traumatic impact (26). Besides, Jamieson *et al*. (13) reported that patients with facial injuries had a high incidence rate of life-threatening craniocerebral injury. Thus, many victims died of craniocerebral injury and were excluded from the study. Soft tissue injuries mainly consist of abrasion and laceration. This is consistent with results of a study that was based on a 10-year survey (24).

In this study, 98 patients sustained 192 facial fractures; 6.12% of the fractures affected the frontal bones, 61.22% the mid face, and 67.35% the mandible. The fractures often occurred in at least two sites. Although most previous studies showed the dominance of mid face fractures in older patients, the distribution of fractures could vary depending on the geographic area and socioeconomic status (9,27). In the midface region, the zygomatic bone was the most frequently involved, followed by maxillary bone. The zygomatic bone could sustain injury because of its prominent positions and anatomic structure susceptible to injury by external forces in the maxillofacial region (28). Our data was in accordance with the studies in Japan and Italy (28,29). In the mandible, fractures were most often observed in the body, followed by symphysis and ramus. These results showed a significant difference compared with the younger groups. It is interesting to note there were only a few condylar fractures, that was different from the studies on younger groups (30). Previous study indicated that the trauma force applied in the symphyseal region often caused indirect fractures of the condyle, with or without fractures in the symphysis (31). However, the elder people often sustained a situation of osteoporosis and mandibular atrophy. It is reported that in an atrophic mandible, a traumatic force, such as falling on the ground or traffic accident, could result in body fractures (28). In patients with 4 or fewer residual teeth, body fractures were observed at a significantly greater rate than in patients with 10 or more residual teeth (28). Furthermore, the rate of body fracture was even greater in an investigation of patients with a totally edentulous mandible (29). We thought the cause might be that the marked reduction of the mandibular height and vascularity could decrease the strength of the mandible, in edentulous or almost edentulous patients.

Maxillofacial injuries may appear over isolation or may be associated with other injuries. Injuries to other sites of the body were observed in 82 patients (65.08%). The most common area of associated injuries was head (56 patients, 44.44%) and extremity (28 patients, 30.16%), along with other organs, such as, chest, abdomen, and spine. The rate was higher than that in previous studies (28,32), probably because most injuries were caused by falling and motor vehicle accidents. Moreover, the rate of associated injuries to other sites of the body was significantly greater in patients injured in traffic accidents. Therefore, other medical departments may be more necessary to the patients injured in traffic accidents. It is reported that life-threatening injuries can sometimes be associated with patients suffering maxillofacial fractures (32). However, we found no deaths in the present study. The reason may be that some patients with these life-threatening injuries could die at the scene or soon after arrival and never reach the maxillofacial department.

Most notably there were no general infections related to the maxillofacial injuries. Only 13patients (10.32%) suffered local infections. During these patients with local infections, most had a medical condition, such as diabetes mellitus. These conditions may increase the risk of infection, especially after surgical operation (33).

To the soft tissue injuries, all the patients were given proper management, such as debridement and suturing. To the facial fractures, open reduction and internal fixation (ORIF) was performed in the majority of the patients. The percentage of operation was higher than other similar studies (22,28). The presence of dental restorations or missing teeth is much more frequent in the geriatric age group and poses a unique challenge when attempting to restore the occlusion and maintain adequate reduction and fixation. Therefore, we have increased the use of ORIF to treat maxillofacial fractures of the elderly in an attempt to alleviate these problems by allowing earlier mobilization. However, the rate of conservative treatment increased in parallel with the increase in patient age. The decision to operate is based on more confounding factors than just severity alone, and includes the preferences of the surgeon, the patient, and the anaesthetist. The elderly patients had a much higher percentage of previous medical problems, defined as one or more of the following: hypertension, heart disease, diabetes mellitus, and respiratory disease, which may increase the risk of operation and anesthesia. Besides, elderly patients also tend to place less focus on aesthetics and more focus on function than younger people.

- Limitation of the study: It is difficult to obtain adequate follow-up information for many of the patients due to their general condition and limited transportation means, as well as reduced life expectancy. Therefore, we cannot make statistical comparisons between groups on the long-term complications or effects of the different treatment methods. Although long-term results in the elderly with major trauma did not show a significant difference, age inevitably causes physiologic changes, such as decreased nutritional status and severe osteoporosis, to the bones and soft tissue that render healing more difficult (27).

In conclusion, our study has shown characteristic features in their etiology, patterns, and treatment modalities of maxillofacial injuries in the elderly patients. Understanding these characteristics could help to promote clinical research to develop more effective treatment and possibly prevent such injuries. Due to the low level of economic development in He’nan, the following three points may be helpful: (1) extensive education to enhance people’s awareness of self-protection and risk identification; (2) different governmental departments should cooperate with each other to improve the safety status of traffic infrastructures and safety facilities in public or working place; (3) increase investment in road construction to increase the measurements such as warning signs, speed limits and specific passages.
